# Effects of *Gigantochloa apus* String Bamboo Fiber Brushes on Heat-Cured Acrylic Resin Plate Surface Roughness

**DOI:** 10.1155/ijod/9054144

**Published:** 2025-01-22

**Authors:** Azkya Patria Nawawi, Asih Rahaju, Annisha Putri Rivawati

**Affiliations:** ^1^Prosthodontic Department, Faculty of Dentistry, Universitas Jenderal Achmad Yani, Cimahi, Indonesia; ^2^Conservative Dentistry Department, Faculty of Dentistry, Universitas Jenderal Achmad Yani, Cimahi, Indonesia; ^3^Faculty of Dentistry, Universitas Jenderal Achmad Yani, Cimahi, Indonesia

**Keywords:** heat-cured acrylic resin plate, string bamboo fiber brush (*Gigantochloa apus*), surface roughness

## Abstract

**Purpose:** The objective of this study was to assess the impact of string bamboo fiber brushes (*Gigantochloa apus*) on the surface roughness of heat-cured acrylic resin plates.

**Materials and Methods:** This study used a pretest and posttest laboratory experiment with a control group design, with data obtained from the average surface roughness values of heat-cured acrylic resin plates after brushing using bamboo silk fiber brushes as the test group and conventional denture brushes as the control group.

**Results:** The Shapiro–Wilk test showed that the data were normally distributed, with a total of 32 samples of heat-cured acrylic resin plates (*p*=0.794). The results of this study found that the average surface roughness values of the anatomical surface of heat-cured acrylic resin plates after being brushed with string bamboo fiber brushes were 0.20 µm and 0.18 µm for the nonanatomical surface. The difference in the average surface roughness of the heat-cured acrylic resin plates, both on anatomical and nonanatomical surfaces, before and after brushing with string bamboo fiber brushes, was 0.0001 (*p*  < 0.05).

**Conclusion:** Bamboo denture toothbrushes exhibit a lower abrasive effect than nylon denture toothbrushes, with the abrasiveness remaining below the threshold requirements established for dental materials.

## 1. Introduction

Removable dental prostheses (RDP) can be removed and reinserted into the oral cavity in patients with tooth loss. The components of an RDP include artificial teeth, clasps, and a base [1–4]. The denture base is the component placed on the soft tissues of the oral cavity, thus facilitating the formation of both pathogenic and nonpathogenic organisms on its surface [5–7]. The material predominantly employed for denture bases is heat-cured acrylic resin, chosen to meet the ideal base material criteria, such as simplicity in manipulation, affordability, nontoxicity, and esthetic appeal [8–10]. Acrylic resin is characterized by its porosity, which influences the surface roughness [10–12]. The acceptable limit for denture base surface roughness is set at 0.2 mm, surpassing which could escalate bacterial adhesion, leading to detrimental outcomes for denture users, such as halitosis, discomfort, compromised esthetics, and mucosal infections, including denture stomatitis [3, 13–16]. Implementing appropriate denture cleaning practices can mitigate these adverse effects.

Hygiene of dentures is a critical concern for both dentists and patients. Dentures can be cleaned using mechanical or chemical methods. Mechanical methods involve brushing the dentures using conventional denture brushes, whereas chemical methods entail immersing them in solutions containing bleaching agents, mineral acids, enzymes, mouthwashes, and disinfectants [17–19]. The mechanical approach is highly recommended by dental professionals for its effectiveness and cost efficiency, despite its drawback of potentially increasing the denture base's surface roughness [20–22]. Surface roughness on the denture base can be caused by abrasion during brushing, related to research conducted by Agnes that showed an increase in the average value of surface roughness on acrylic plates after brushing [23]. The abrasion occurring during denture cleaning can be caused by the hardness of the brush bristles, brushing technique, and frequency of use [20]. An ideal brush for cleaning dentures should be equipped with soft bristles to reduce wear on the denture surface. Conventional denture brushes currently used are made of nylon fibers, where previous research has shown that this material still causes wear on the surface [20, 22, 23].

An effort that can be made is to determine the appropriate denture cleaning tool by selecting soft brush bristles that do not cause wear on the acrylic resin surface [23]. One material that can be considered as an alternative for brush bristles is natural fibers. Bamboo is a natural fiber that can be considered as an alternative material for denture brush bristles because of its availability, environmental friendliness, and many advantages. Bamboo can thrive in areas with high rainfall, and there are 60 species of bamboo in Indonesia, making it very easy to find bamboo in Indonesia, for example, in the Cimahi area [24]. One type of bamboo commonly found in Cimahi is string bamboo (*Gigantochloa apus*) [25]. The large population and favorable characteristics of string bamboo are reasons why this plant is widely utilized by the community. String bamboo is considered to produce fibers that are strong, smooth, and flexible [26–30]. Bamboo contains parenchyma, which causes it to have water absorption properties. The water absorption property determines the hardness and strength of the fibers produced by bamboo, while each layer of bamboo has different water absorption capabilities [27]. Therefore, the fiber properties produced by each layer of bamboo will indeed differ, requiring special attention to determine whether the fibers produced are suitable as an alternative material for denture brush bristles and do not cause wear. This can be assessed by their effect on the surface roughness of heat-cured acrylic resin plates, as the surface roughness property can determine the durability and both positive and negative impacts on denture users [28, 31, 32].

Research on the effect of string bamboo fiber brushes (*Gigantochloa apus*) on the surface roughness of heat-cured acrylic resin plates has not been conducted previously. Based on the background described above, researchers are interested in investigating the effect of bamboo fiber brushes obtained from plantations in Cimahi City on the surface roughness of heat-cured acrylic resin plates.

Based on this, it was hypothesized that bamboo toothbrushes can provide a smaller abrasive effect than nylon denture toothbrushes.

## 2. Materials and Methods

**Figure 1:**
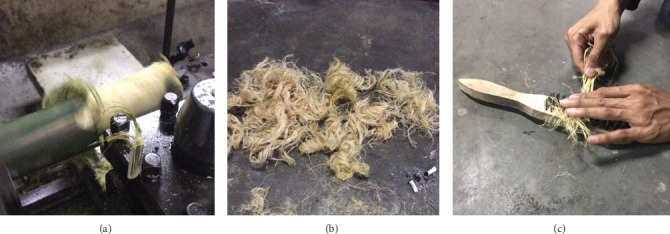
Fabrication of bamboo fiber brushes. (A) Turning process. (B) Bamboo fiber results from turning process. (C) Smoothing fibers using a wire brush.

**Figure 2:**
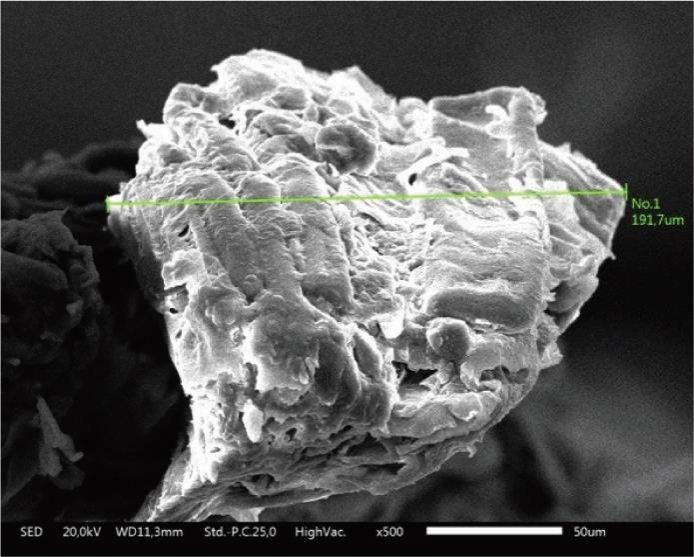
SEM result at 500× magnification. Average diameter of bamboo fiber about 191.7 μm.

**Figure 3:**
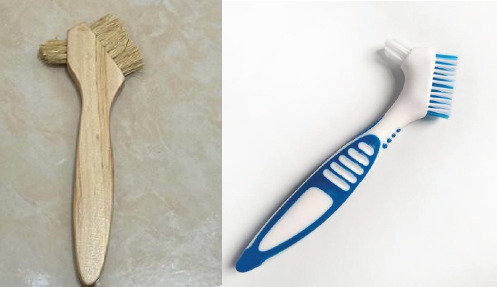
Bamboo denture brush and nilon denture brush.

The research procedure conducted in this study consisted of three stages: making string bamboo fiber brushes, making heat-cured acrylic resin plates, and testing surface roughness. String bamboo fiber brushes were made by turning the bamboo stem to a thickness of 1.5 mm to obtain smooth fibers, which were sorted using a wire brush and standardized in size using scissors. (Figure 1).To ensure an appropriate size for each fiber, SEM tests were conducted (Figure 2). The final results for the bamboo brush are shown in Figure 3.

**Figure 4:**
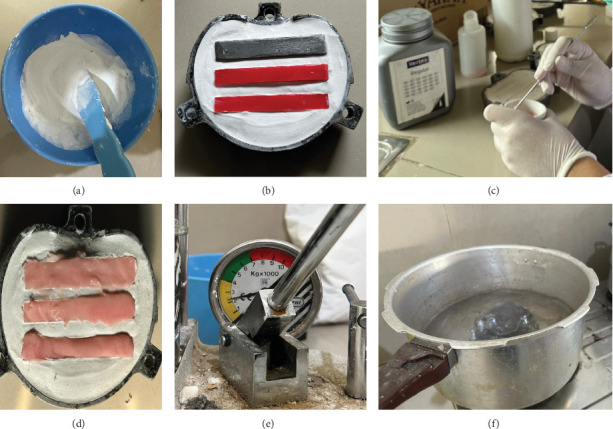
Fabrication of the acrylic resin plates. (A) Gips plaster. (B) Molding the acrylic. (C–E) Packing. (F) Curing process.

A mold for heat-cured acrylic resin plates was created using a metal master model (75 mm × 10 mm × 3 mm, ISO 527-1:2019). Acrylic resin (ADM Heat Curing, England) with a monomer:polymer ratio of 2 g polymer to 1 mL monomer was stirred until it reached the dough stage, and the flask was closed and subjected to 1000 psi pressure using a press machine. After removing and opening the flask, excess resin was cleaned with a lecron. The flask was then reclosed, pressed at 2200 psi, and secured with bolts to ensure tight sealing. The curing process for the acrylic resin was performed using a pot containing boiling water (Figure 4). The flask was immersed in the pot for 30 min, then removed, and allowed to cool to room temperature. The plate was then polished.

**Figure 5:**
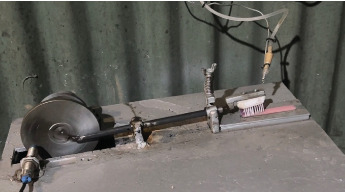
Brushing simulation machine test.

Surface roughness testing was conducted using a surface roughness tester (AR-131A+, Aermanda, China) and heat-cured acrylic resin plates before and after the treatment. The treatment test was performed by brushing the heat-cured acrylic resin plates using a brush simulation machine. The treatment test was conducted on two groups: string bamboo fiber brushes as the test group and conventional denture brushes as the control group. Heat-cured acrylic resin plates were placed on a flat surface, and a toothbrush was placed on the head of the brush simulation machine. The pressure load was set at 200 g to represent the force during brushing, and the speed was set at 365 rpm for 50 min, with 17,800 rotations representing denture brushing for a year. Brushing of the heat-cured acrylic resin plates was performed on both the anatomical and nonanatomical surfaces (Figure 5).

**Figure 6:**
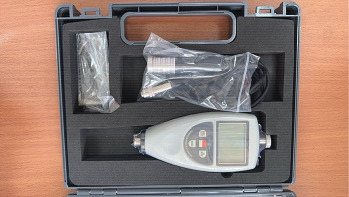
Surface roughness tester AR-131A+.

Surface roughness testing was carried out by first placing the acrylic resin plate on a flat surface and then turning on the surface roughness tester by pressing the on button. The surface of the acrylic resin plate was divided into three parts, and the needle was directed to move along the surface at a distance of 1–2 mm. Measurements were taken three times on the divided surface, and the measurement results displayed on the surface roughness tester were in micrometers (µm). The surface roughness values obtained from the three measurements were then summed and averaged to obtain the average roughness (Ra) value (Figure 6).

The data analysis in this study began with a normality test using the Shapiro–Wilk test (*n* < 50). The results indicated that the data were normally distributed, and a paired *t*-test was conducted to determine the difference in the average surface roughness values of the heat-cured acrylic resin plates before and after treatment with string bamboo fiber brushes. Additionally, an independent *t*-test was used to compare the average surface roughness values of heat-cured acrylic resin plates brushed with string bamboo fiber brushes and conventional denture brushes.

## 3. Results

Normality tests were conducted on the data obtained using the Shapiro–Wilk test. The results of the normality test showed that the data were normally distributed (*p*=0.794), and a paired *t*-test was used to determine the effect of the bamboo fiber brush on the surface roughness of the heat-cured acrylic resin plate before and after brushing.

The data from this investigation demonstrate the average surface roughness values on anatomic and nonanatomic surface of heat-cured acrylic resin plates before (anatomic surface = 0.18 ± 0.012; nonanatomic surface = 0.14 ± 0.007) and after brushing with string bamboo fiber brushes (anatomic surface = 0.20 ± 0.015; nonanatomic surface = 0.18 ± 0.015) (Table 1), as well as the comparison of average surface roughness values on anatomic and nonanatomic surface of heat-cured acrylic resin plates between the use of string bamboo fiber brushes (anatomic surface = 0.20 ± 0.015; nonanatomic surface = 0.18 ± 0.015) and conventional denture brushes (anatomic surface = 0.23 ± 0.015; nonanatomic surface = 0.20 ± 0.012) (Table 2). The surface roughness values of the acrylic resin plates were measured using a surface roughness tester, and the results are expressed in micrometers (µm). Measurements were conducted in triplicate, and the results were aggregated and averaged to obtain the Ra value. The assessment of the surface roughness of heat-cured acrylic resin plates yielded diverse results.

Table 1 presents the results of the independent *t*-test, with a *p*-value <0.05 (0.0001) indicating a statistically significant difference in the average surface roughness of heat-cured acrylic resin plates, both on anatomical and nonanatomical surfaces, before and after brushing with string bamboo fiber brushes. This finding suggests that the use of string bamboo fiber brushes increases the surface roughness of heat-cured acrylic resin plates.

Table 2 illustrates the results of the paired *t*-test, with a *p*-value <0.05 (0.0001) indicating a statistically significant difference in the average surface roughness between the anatomical and nonanatomical surfaces of heat-cured acrylic resin plates after brushing with string bamboo fiber brushes and conventional denture brushes.

## 4. Discussion

In this study, the results of the surface roughness test presented in Table 1 indicate that the average surface roughness values of the anatomical surface of heat-cured acrylic resin plates after brushing with string bamboo fiber brushes were 0.20 µm, and for the nonanatomical surface, it was 0.18 µm. Surface roughness in restorative materials arises from several undesirable factors such as friction forces, scratches, and chemical reactions [33, 34]. Acrylic resin, used as a denture base, possesses porosity characteristics that can influence surface roughness. Higher surface roughness values make denture bases difficult to clean, thereby facilitating bacterial adherence to the acrylic plate surface [11, 35].

In this research, mechanical cleaning of dentures was performed using brushes without toothpaste, correlating with Kurniawan's study, which stated that cleaning dentures with brushes without toothpaste yields better average surface roughness values for heat-cured acrylic resin plates compared to using toothpaste [23]. The simulation test in this research was carried out using string bamboo fiber brushes and conventional denture brushes for 50 min at a speed of 365 rpm and a pressure load of 200 g to represent a year's worth of denture brushing [20, 36]. The change in average surface roughness values of heat-cured acrylic resin plates after brushing is presumed to arise from friction forces, as well as the strength and hardness of the fibers used [20, 21].

The findings of this study revealed a significant difference between the surface roughness of acrylic resin plates brushed using conventional denture brushes and string bamboo fiber brushes. The acceptable surface roughness threshold in dentistry is 0.20 µm; in this study, the average surface roughness value of the anatomical surface after brushing with conventional denture brushes was 0.23 µm (>0.20 µm), meaning it exceeded the acceptable threshold for surface roughness in dental materials. This is related to Livia Rukmana's research, suggesting that the surface roughness value of heat-cured acrylic resin plates used as denture bases should not exceed 0.20 µm, as surpassing this limit would ease microorganism settlement and create a detrimental oral environment [5, 15, 16, 37].

The strength of a material can be assessed on the basis of its abrasion level. The tensile strength of acrylic resin is ~50 MPa, indicating a low tensile strength that can cause abrasion or fractures under pressure [10, 38, 39]. Abrasion arises from the friction of a material's force, causing particles to wear away [18, 40]. The abrasion occurring during the brushing of dentures results in surface wear, thereby altering the average surface roughness values of heat-cheat-curedic resin plates after brushing with either string bamboo fiber brushes or conventional denture brushes [18, 22, 23]. Bamboo contains parenchyma, which leads to its water content and emergence of water absorption properties. The higher the parenchyma and lower the fiber density, the greater the water absorption and content in the bamboo. For instance, the base of bamboo has the highest amount of parenchyma and lowest fiber density. This results in the bamboo base exhibiting higher water absorption and content properties [16]. Bamboo fibers have a water absorption capacity of 30%–31.91%, whereas nylon fibers have a water absorption capacity of 4% [41–45]. The greater a material's capacity for water absorption, the lower its strength and hardness; hence, nylon fibers will possess higher strength and hardness compared to bamboo fibers [46].

The strength and hardness of brush bristles can influence the risk of wear on acrylic resin plates, which will increase the surface roughness values [20, 22]. Based on the strength and hardness of the bristles, the use of string bamboo fibers will result in lower wear than the use of nylon fibers. Therefore, in this study, the average surface roughness values of heat-cured acrylic resin plates brushed with string bamboo fiber brushes were lower than those brushed with conventional denture brushes. This finding is supported by the research of Saishree, which states that the use of bamboo fiber brushes leads to lower wear than that caused by nylon fiber brushes [23, 40, 47].

The primary strength of this study lies in its novel investigation of bamboo as a potential fiber for denture toothbrushes, with the results indicating that bamboo fiber may be considered a viable option for this purpose. However, a limitation of this study is the absence of microbiological analysis regarding the effectiveness of cleaning bamboo denture toothbrushes. Future studies should address this gap by examining the efficacy of cleaning bamboo denture toothbrushes from a microbiological perspective.

## 5. Conclusion

The research findings indicate that bamboo denture toothbrushes exhibit a lower abrasive effect than nylon denture toothbrushes, with abrasiveness remaining below the threshold requirements established for dental materials.

## Figures and Tables

**Table 1:** The effect of string bamboo fiber brushes on the surface roughness of heat-cured acrylic resin plates.

Type of heat-cured acrylic resin plate surface	Average surface roughness (Ra)		*p* value
	Before brushing	After brushing	
	*N* = 16	*N* = 16	
Anatomical surface			
Mean ± standard	0.18 ± 0.012	0.20 ± 0.015	0.0001*⁣*^*∗∗*^
Nonanatomical surface			
Mean ± standard	0.14 ± 0.007	0.18 ± 0.015	0.0001*⁣*^*∗∗*^

*⁣* ^*∗∗*^ *p* < 0.05.

**Table 2:** Difference in surface roughness of heat-cured acrylic resin plates brushed using string bamboo fiber brushes and conventional denture brushes.

Type of heat-cured acrylic resin plate surface	Average surface roughness after brushed (Ra)		*p* value
	String bamboo fiber brush	Conventional denture brush	
	*N* = 16	*N* = 16	
Anatomical surface after			
Mean ± standard	0.20 ± 0.015	0.23 ± 0.015	0.0001*⁣*^*∗∗*^
Nonanatomical surface after			
Mean ± standard	0.18 ± 0.015	0.20 ± 0.012	0.0001*⁣*^*∗∗*^

*⁣* ^*∗∗*^ *p* < 0.05.

## Data Availability

The data used to support the findings of this study are included within the article and available from the corresponding author (Azkya Patria Nawawi) upon reasonable request.
